# Integrating blue: How do we make nationally determined contributions work for both blue carbon and local coastal communities?

**DOI:** 10.1007/s13280-022-01723-1

**Published:** 2022-05-03

**Authors:** Amrit Melissa Dencer-Brown, Robyn Shilland, Daniel Friess, Dorothée Herr, Lisa Benson, Nicholas J. Berry, Miguel Cifuentes-Jara, Patrick Colas, Ellyn Damayanti, Elisa López García, Marina Gavaldão, Gabriel Grimsditch, Adam P. Hejnowicz, Jennifer Howard, Sheikh Tawhidul Islam, Hilary Kennedy, Rahma Rashid Kivugo, Joseph K. S. Lang’at, Catherine Lovelock, Ruth Malleson, Peter I. Macreadie, Rosalía Andrade-Medina, Ahmed Mohamed, Emily Pidgeon, Jorge Ramos, Minerva Rosette, Mwanarusi Mwafrica Salim, Eva Schoof, Byomkesh Talukder, Tamara Thomas, Mathew A. Vanderklift, Mark Huxham

**Affiliations:** 1 Edinburgh Napier University, 9 Sighthill Court, Edinburgh, EH11 4BN Scotland; 2 School of Applied Sciences, Edinburgh Napier University, Edinburgh, EH11 4BN Scotland; 3 Department of Geography, National University of Singapore, Singapore, Singapore; 4 NUS Centre for Nature-Based Climate Solutions, National University of Singapore, Singapore, Singapore; 5 Global Marine and Polar Program, IUCN, Gland, Switzerland; 6 The Centre for Environment, Fisheries and Aquaculture Science (Cefas), Pakefield Road, Lowestoft, NR33 0HT Suffolk UK; 7 The Landscapes and Livelihoods Group LLP, Edinburgh, UK; 8 CATIE - Centro Agronómico Tropical de Investigación y Enseñanza, Turrialba, 30501 Costa Rica; 9 Conservation Finance Africa Field Division - Conservation International, Ndege Road, Nairobi, Kenya; 10 Faculty of Forestry and Environment, IPB University, Bogor, 16680 Indonesia; 11 CINVESTAV - Laboratorio de Producción Primaria, Recursos del Mar, Centro de Investigación y de Estudios Avanzados del Instituto Politécnico Nacional – Unidad Mérida, Carretera Antigua a Progreso Km 6, CP 97310 Mérida, Yucatán México; 12 Resiliencia Azul (NPO), Mogi das Cruzes, Mexico; 13 Ubá Sustainability Institute - Blue Carbon Hub, Marseille, France; 14 United Nations Environment Programme, UN Avenue, PO Box 67578, Nairobi, Kenya; 15 School of Engineering, Newcastle University, Newcastle upon Tyne, UK; 16 Department of Biology, University of York, York, UK; 17 Blue Carbon Program, Conservation International, 2011 Crystal Drive, Suite 600, Arlington, VA 22202 USA; 18 Institute of Remote Sensing and GIS, Jahangirnagar University, Dhaka, 1342 Bangladesh; 19 School of Ocean Sciences, Bangor University, Wales, LL59 5AB UK; 20 Mikoko Pamoja Community Base Organization, P.O. BOX 178-80404, Msambweni, Kenya; 21 Kenya Marine and Fisheries Research Institute, P. O. Box 81651-80100, Mombasa, Kenya; 22 School of Biological Sciences, The University of Queensland, St Lucia, QLD 4072 Australia; 23 University College London, 14 Taviton Street, London, WC1H 0BW UK; 24 Centre for Integrative Ecology, School of Life and Environmental Sciences, Deakin University, Burwood Campus, Burwood, VIC 3125 Australia; 25 Center for Oceans, Conservation International, 2011 Crystal Drive, Suite 500, Arlington, VA 22202 USA; 26 Institute for Land, Water and Society, Charles Sturt University, PO Box 6087, South Bunbury, WA 6230 Australia; 27 Vanga Blue Forest Community Based Organization, P.O Box 115-80402, Lungalunga, Kwale County Kenya; 28 Plan Vivo Foundation, Thorn House, 5 Rose Street, Edinburgh, EH2 2PR UK; 29 Dahdaleh Institute for Global Health Research, York University, Toronto, ON Canada; 30 International Ocean Policy, Global Policy and Government Relations, Conservation International, 2011 Crystal Drive, Suite 600, Arlington, VA 22202 USA; 31 CSIRO Oceans & Atmosphere, Indian Ocean Marine Research Centre, Crawley, WA 6009 Australia

**Keywords:** Blue carbon, Conservation, Local livelihoods, Nature-based solutions, NDCs, Sustainability

## Abstract

Blue Carbon Ecosystems (BCEs) help mitigate and adapt to climate change but their integration into policy, such as Nationally Determined Contributions (NDCs), remains underdeveloped. Most BCE conservation requires community engagement, hence community-scale projects must be nested within the implementation of NDCs without compromising livelihoods or social justice. Thirty-three experts, drawn from academia, project development and policy, each developed ten key questions for consideration on how to achieve this. These questions were distilled into ten themes, ranked in order of importance, giving three broad categories of *people*, *policy & finance*, and *science & technology*. Critical considerations for success include the need for genuine participation by communities, inclusive project governance, integration of local work into national policies and practices, sustaining livelihoods and income (for example through the voluntary carbon market and/or national Payment for Ecosystem Services and other types of financial compensation schemes) and simplification of carbon accounting and verification methodologies to lower barriers to entry.

**Supplementary Information:**

The online version contains supplementary material available at 10.1007/s13280-022-01723-1.

## Introduction

The UNFCCC *Paris Agreement* commits signatories to ‘pursue efforts’ to limit the increase in global average temperature to 1.5 °C. Achieving this requires a rapid decarbonisation of the global economy. However, decarbonisation alone will not be sufficient; IPCC scenarios for limiting global temperature rise to 1.5 °C also require the removal of increasing amounts of carbon dioxide from the atmosphere (IPCC 2013). This will rely in part on nature-based solutions (NbS) to conserve and expand natural carbon sinks, delivering climate change mitigation benefits along with co-benefits to society and biodiversity (IUCN 2020). Due to their efficiency in the capture and storage of carbon (relative to terrestrial ecosystems), mangroves, seagrass and tidal marshes—the so-called Blue Carbon Ecosystems (BCEs)—are amongst the most important habitats for climate change mitigation and adaptation (Macreadie et al. 2019). Hence, protection and restoration of BCEs are increasingly recognised as important forms of NbS for achieving climate policy initiatives at local and global scales (Seddon et al. 2020, 2021). Protection and restoration of BCEs offer potentially high returns on investment; Stuchey et al. (2020) report that mangrove conservation and restoration alone could deliver US$0.2 trillion over a 30-year period, delivering a high benefit–cost ratio of 3:1. One hundred and fifty-one countries around the world contain at least one BCE and 71 contain all three (Blue Carbon Initiative 2019), hence there are compelling arguments and multiple opportunities for nations to incorporate BCEs into climate policy.

Nationally Determined Contributions (NDCs) represent the primary mechanism for meeting the global ambitions of the Paris Agreement. Here, countries commit to nationally appropriate actions to mitigate against and adapt to climate change (Article 4.2 Paris Agreement; UNFCCC 2020). NDCs operate on a ratcheting five-year cycle, with each round of public submissions advancing in ambition relative to the previous round (Article 4.3 Paris Agreement). Hence, in theory, the NDC process offers a flexible and transparent mechanism to accelerate progress towards global climate change goals by allowing countries to focus on sensible national priorities, permitting leading nations to set stretching targets and exposing missed commitments and inadequate goals to public scrutiny.

In the first NDC submissions of 2015, 59 countries included coastal ecosystems in their adaptation responses whilst 28 included them as part of mitigation strategies (Herr and Landis 2016). The NDC Partnership (a global coalition of governments and institutions supporting the implementation of NDCs) had received 60 requests from 17 countries for support for NDC implementation plans related to ‘oceans and coasts’, ahead of the second round of submissions in 2020/21 (NDC Partnership 2019). Of these, 13 countries had already included coastal ecosystems in their previous NDC submissions; however, explicit mention of and concrete targets for BCEs remains limited (NDC Partnership 2019). There is therefore considerable scope for the inclusion, enhancement and conceptualisation of BCEs into many more NDCs, including those in which they are already mentioned. Due to delays caused by the Covid-19 pandemic it is possible that some of these opportunities could be realised in this second round of NDC submissions but will certainly be available during the next phase of 2025.

General guidance on developing NDCs includes the importance of community consultation (e.g. Fransen et al. 2017; NDC Partnership 2020). Some NbS projects, including those involving mangroves, have been criticised for failing to respect the rights and agency of local people and for enforcing ‘fortress conservation’ models of protection (e.g. Beymer-Farris and Bassett 2012). Working in partnership with communities is important for many reasons, but three stand out as especially significant for BCEs. First, environmental justice requires that those most affected by climate change and most vulnerable to its impacts (both short and long-term) are central to responses to mitigate and adapt to those changes. Second, BCEs are typically contiguous with human communities who are heavily dependent upon them, hence are usually best understood as socio-ecological (rather than only biological) systems. Third, the knowledge that local communities have, for example about past distributions and diversity, may be vital for effective conservation and restoration activities. Changes in governance regimes associated with BCEs can disproportionately affect the most vulnerable people for good or ill (Fortnam et al. 2021), and management models that are not consented to and supported by local people are likely to fail and to increase development inequalities (Nunan et al. 2021)*.*

**Fig. 1:**
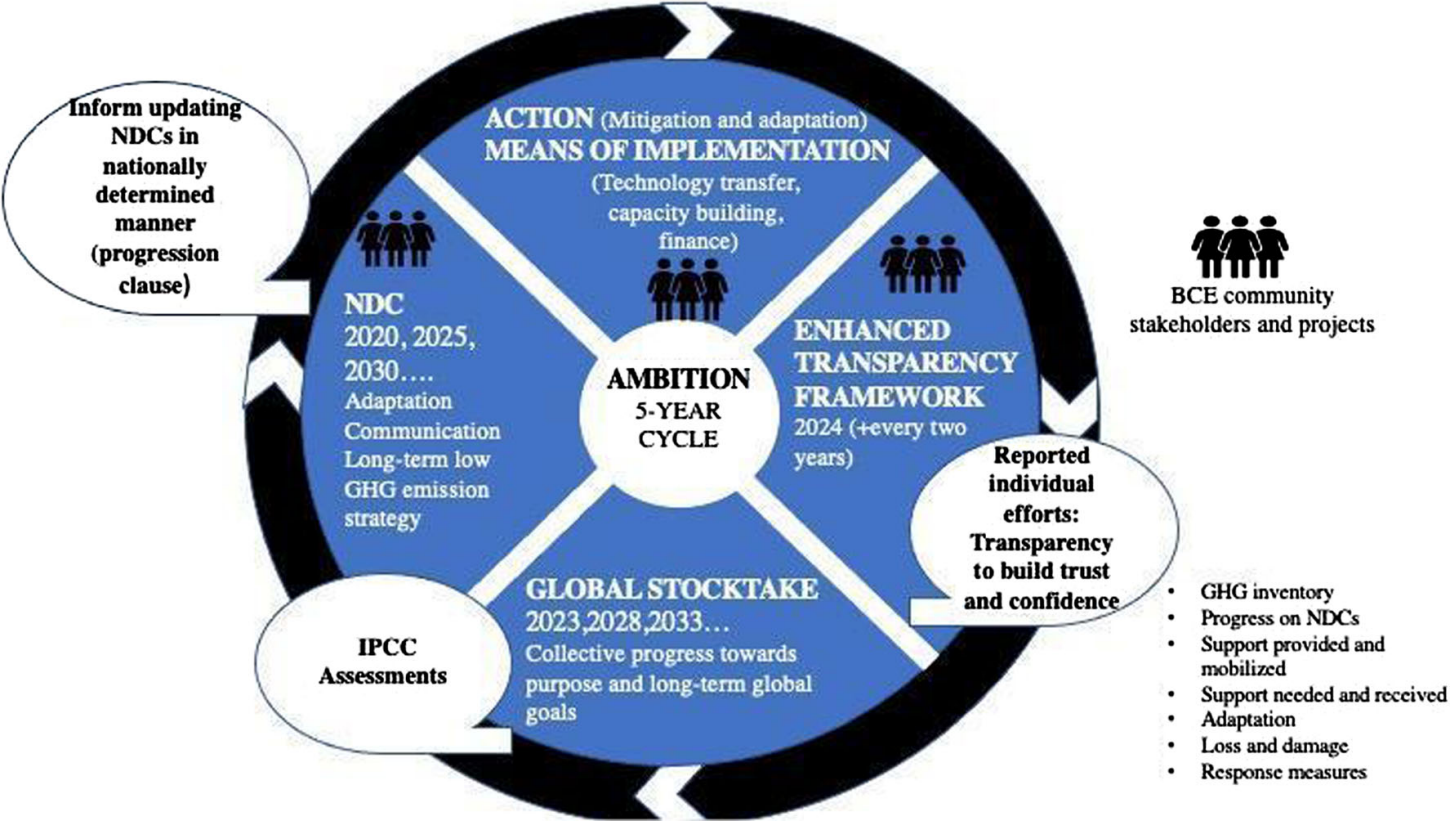
Blue NbS and the NDC ratchet mechanism with potential points in the NDC cycle for community engagement (adapted from UNFCCC Secretariat in Von Unger et al. 2020)

To be just and successful, the NDC consultation process must involve the local communities themselves in the planning and implementation stages. Several opportunities and interventions are possible to allow NDCs to do this (Fig. 1). For example, community participation may feature during implementation of projects, specifically with capacity building, the enhanced transparency framework (which reports on individual efforts of countries) and NDC submissions, which are due every five years. Without community involvement in these areas, it is likely that information for submissions would not be complete.

Here, we identify some of the key issues and challenges that are facing communities, policy makers, managers and other stakeholders when considering how best to use the opportunity presented by NDCs to achieve effective and socially just BCE protection and restoration.

## Horizon scan methodology

**Fig. 2:**
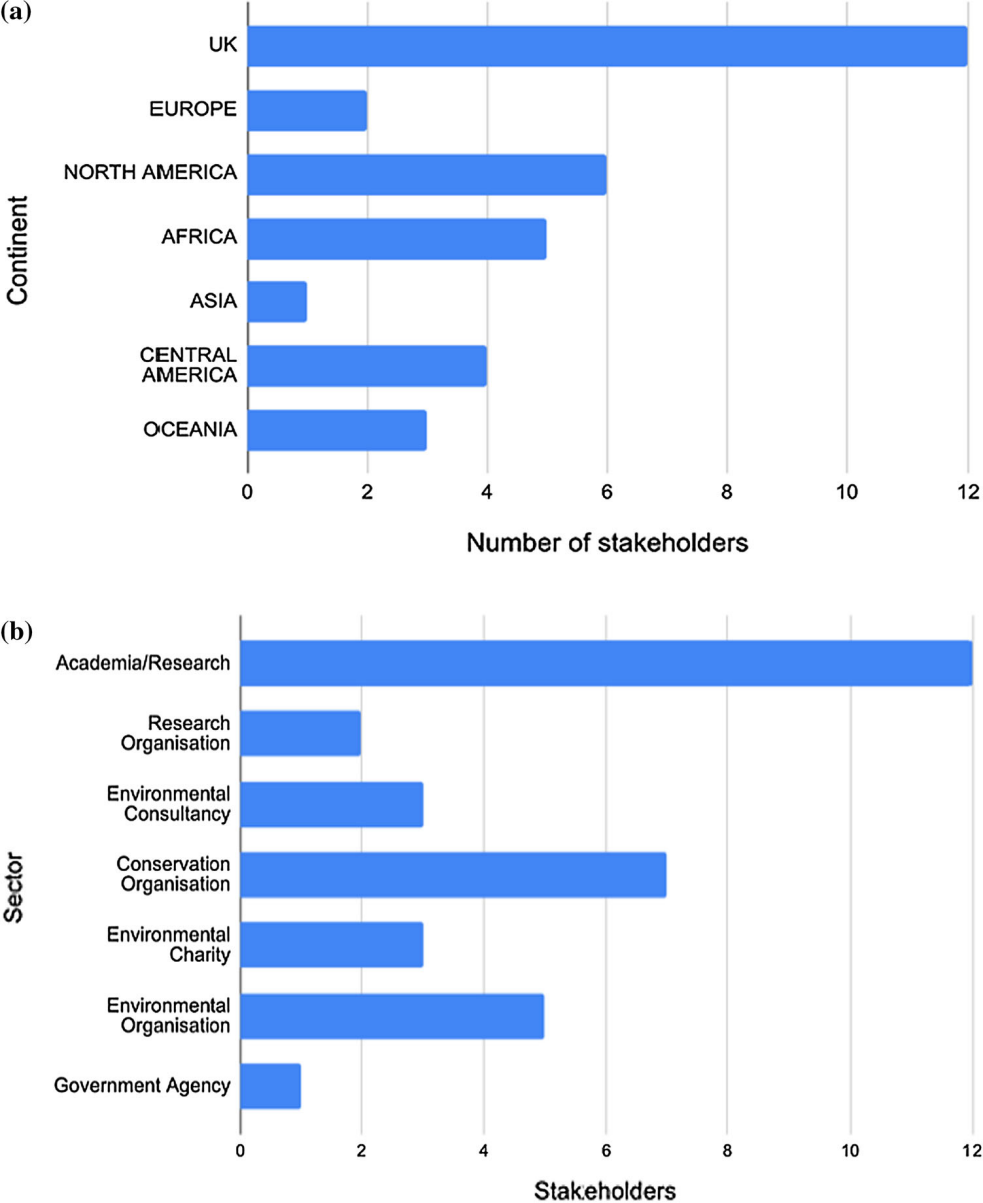
**a** Geographic distribution of stakeholders and **b** Field of work of stakeholders

Horizon scans identify gaps, threats and opportunities which have not been addressed in detail before, with the aim of outlining future priorities in the field (Sutherland and Woodroof 2009; Sutherland et al. 2013; Cook et al. 2014). Through existing networks and a literature search, we identified and contacted 50 experts in the field of blue carbon. Of these, 33 responded, drawn from a wide geographic distribution and range of backgrounds, including academia, conservation, government agencies and project development (including project officers based on site at two ongoing BCE projects). (Fig. 2a and b). We acknowledge that private stakeholders were not included (there was no response from contacting these stakeholders), and results are weighted towards those in research/academia.

Stakeholders were asked the following: “What are your top ten fundamental questions for the incorporation of carbon (in particular blue carbon) into NDCs at a community-level?”. We received a total of 197 questions (some stakeholders grouped and worked together on question development and some submitted fewer than ten questions), which were sorted qualitatively into main themes by three team members using an iterative approach (following the progressive refinement of qualitative themes as described by Williams and Moser 2019). Each question was categorised independently by one individual into self-defined themes. These categorisations were then sense-checked with the other two team members, with questions moved between themes if required following discussion. Theme titles were then confirmed following discussion, with a final list of ten themes and 111 questions emerging. The initial questions, using verbatim submitted language, were then shared again with all stakeholders using survey software (Survey Hero- available from surveyhero.com), grouped within the emergent themes; stakeholders were asked to rank these questions in importance for the successful incorporation of BCEs into NDCs with full engagement of local communities. The top ranked question under each theme then formed the overarching research question reported here. Stakeholders were then allocated themes according to their specific areas of expertise, with the remit of endorsing, editing or re-writing preliminary draft text written by the core team. Stakeholders were provided a list of all questions for each theme. For a list of all questions see Appendix S1.

## Results

**Fig. 3:**
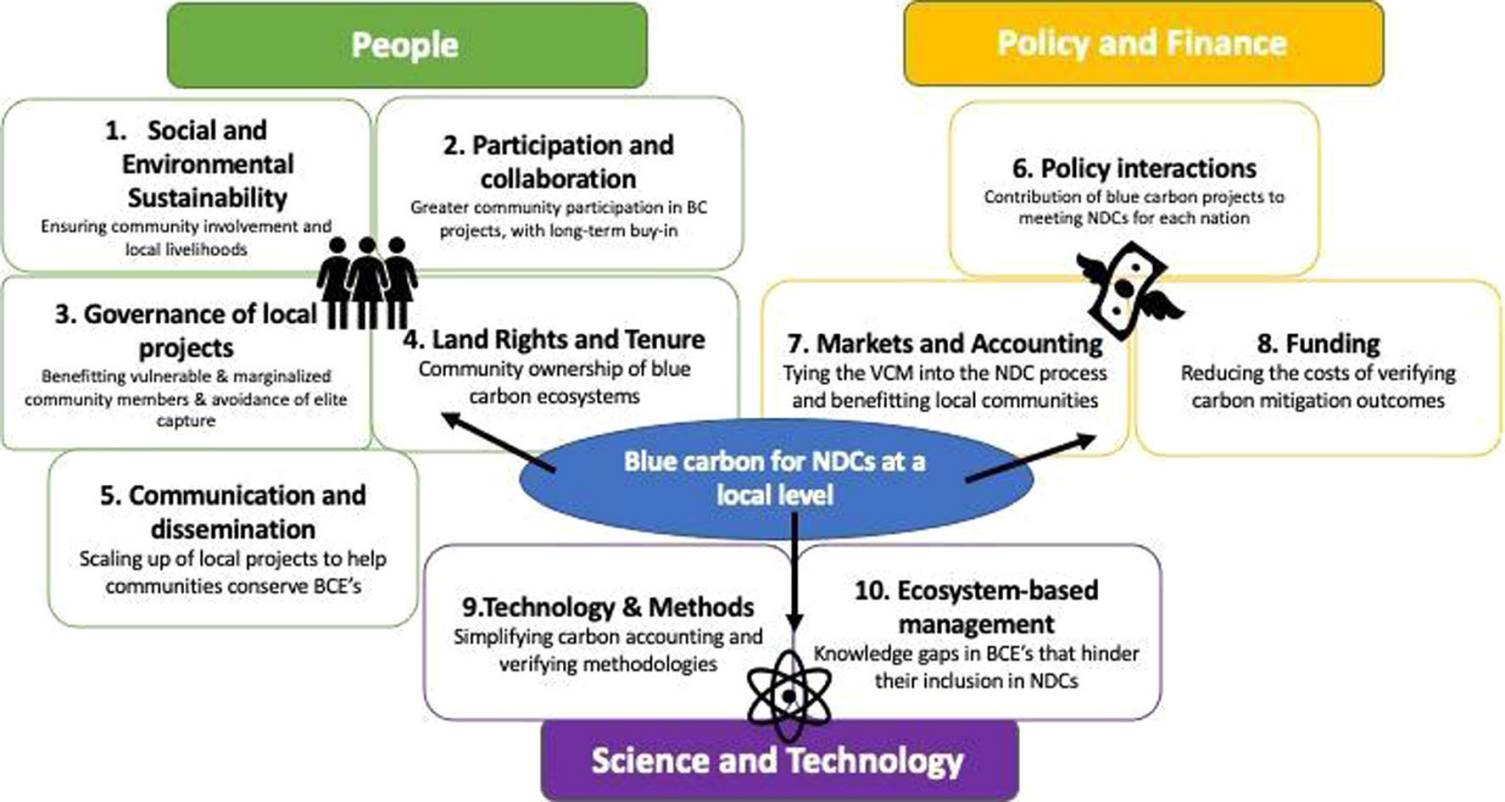
Ten key themes and questions emerging from the scan in three broad areas of people, policy and funding and science and technology

The ten emergent themes fell into the three broad areas of (1) people, (2) policy and finance and (3) science and technology (Fig. 3).

### People

#### Theme 1: Environmental and social sustainability

##### How do we safeguard the sustainability of local livelihoods when implementing and operating BC projects?

**Fig. 4:**
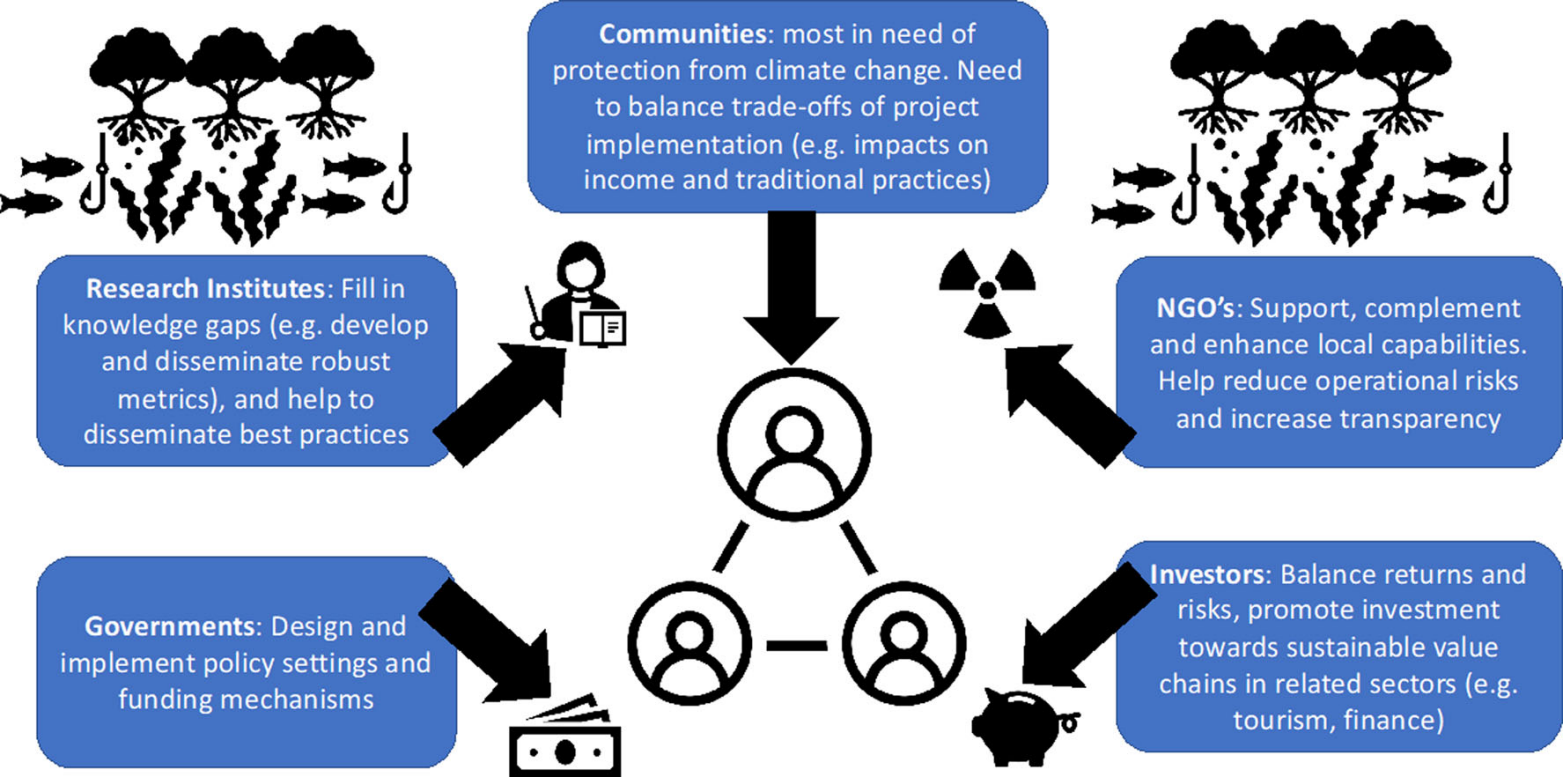
Stakeholders in blue carbon activities, with communities at the heart of the process. Adapted from Vanderklift et al. (2019)

To ensure long-term support from communities for the conservation of BCEs, livelihoods must be sustained, and alternative income generation (AIG) created, where unsustainable use of BCEs threaten their conservation e.g. excessive logging of mangroves for firewood and timber (e.g. Badola et al 2012). Using carbon credits for blue carbon offsetting has been successful in supporting community development projects such as Vanga Blue Forest in Kenya (ACES 2021). Here, the opportunity costs of forest protection (foregoing mostly illegal and small-scale cutting) are outweighed by carbon income, with the individuals most affected (such as cutters) compensated through new opportunities (for example to act as forest scouts). Co-management rights to the forest under national legislation and strong collaboration between the community, the government, NGOs and research institutions have been key to the sustainability of this project; successful BCE projects will typically need good multi-stakeholder collaboration (Fig. 4).

Using Payments for Ecosystem Services (PES—for example the sale of carbon credits) is one approach to help with long-term project funding and to compensate for conservation impacts on livelihoods (Van Hecken et al. 2015; Shapiro-Garza et al. 2020). Demand is growing; trading on the voluntary market increased by 6% between 2018 and 2019, with particularly strong demand for credits related to NbS, which saw a 30% increase in price (Forest Trends’ Ecosystem Marketplace 2020). Interventions that improve gender equity and biodiversity conservation together with mitigation actions are becoming more attractive than projects with a single purpose (Herr et al. 2015) and new forms of crediting that recognise these broader aims, such as Sustainable Development Goal credits, are emerging (Verra 2020). However, all such funding, dependent as it is on global relationships between polluters (or ‘funders’), local stewards and ecosystems, must be regarded as inherently uncertain; it is incumbent, therefore, on project teams to look for ways in which funding can help establish or reinforce less fragile sources of livelihoods and also secure alternative long-term financial sustainability mechanisms.

#### Theme 2: Participation and collaboration

##### How do we promote greater participation of local communities in blue carbon projects, when their most pressing needs are related to immediate livelihoods and infrastructure?

Local perceptions of project legitimacy, derived at least in part from participation, are widely acknowledged to be critical to sustained success in community-based conservation, particularly in PES (Wunder et al. 2018; Wells et al 2020). Frameworks for just and sustainable community engagement with BCE projects are emerging (e.g. The Commonwealth Blue Charter 2021) and include the Code of Conduct for Blue Carbon (Bennett et al. 2017; Blue Carbon Code of Conduct), developed by 96 stakeholder institutions and individuals. This represents an explicit acknowledgement of the importance of community rights and participation and shows a unity of purpose amongst the Blue Carbon community in learning from mistakes made in terrestrial ecosystems. Whilst arguments for community participation are just as strong in terrestrial as in BCE projects, the latter differ in emerging later and hence benefiting from other’s experience. Hence, the intention is clear, but achieving it requires patience, skill and funding.

Multiple approaches can be used to create socially inclusive and participatory governance; of special relevance to BCEs is the creation of networks of locally managed marine areas (LMMAs), or responsible fishing areas, involving local people in community monitoring and compliance. These can provide protection for the long-term storage of Blue Carbon and buy-in from local communities (Vierros 2017; Moraes 2019). Local education campaigns may also be particularly important for BCEs since their benefits (particularly those associated with ‘hidden’ habitats such as subtidal seagrass) may be less obvious to people than those from terrestrial forests. Whilst direct financial or livelihood benefits are likely to be an important predictor of the long-term success of BCE community projects, evidence from terrestrial PES shows that non-financial incentives, including a sense of community pride and ownership, can also be highly influential (Pascual et al 2014). However, participation of the most marginalised people in governance processes is likely to need explicit recognition and financial support, as part of a procedural justice approach (Theme 3).

#### Theme 3: Governance of local projects

##### How can we distribute benefits from blue carbon projects within communities in a manner that benefits the most vulnerable and marginalised members and avoids elite capture?

In general, BCE projects that are perceived to have local legitimacy and that build on and respect de facto governance models are more likely to succeed (Nunan et al. 2021). However, this on its own does not ensure such projects will enhance equity; for example, existing inequalities may be perpetuated and upheld if they are rooted in cultural norms, resulting in elite capture of benefits by those with the most resources and power (Staddon et al. 2015). Implementation of the NDCs should go beyond recognition of local agency to include explicit social justice aims; for example, the NDC Partnership has created a strategy which aims to integrate gender equality into national plans (NDC Partnership 2019). Hence, an explicit commitment to procedural justice, defined as the concept of fair social processes and procedures in decision-making (Tyler and Lind 2002), is needed to help achieve these broader aims of inclusion. Wood et al. (2018) propose a useful six-step approach to manage power in Community-Based Climate Change Development projects (CB-CCD), which involves explicit attempts to avoid domination by one or more powerful groups and ways to ensure the vulnerable are empowered and can voice grievances (Table 1).

**Table 1:** Six steps to manage power in community-based climate change development projects (CB-CCD) adapted from Wood et al. (2018)

Step	Approach	References
Co-producing power analyses	Underpin interventions co-produced by local people and others who have insights into local contexts. These should proceed in a reflexive* way, which reduces subversion by powerful community actors *reflexive meaning to look at one’s own judgements and belief system	Barnaud and Van Paassen. (2013), Wood et al. (2018)
Reducing opportunities for domination	Reduce chances for powerful local actors to dominate and manipulate projects by introducing them through multiple fora with few exit and entry barriers	Wood et al. (2018)
Identifying enabling factors engaging the most vulnerable	Using results of power analyses to identify enabling factors which help overcome resource barriers for the most vulnerable. Providing immediate-term benefits which offset costs of foregoing alternative livelihoods	Wood et al. (2018)
Taking steps to reconcile world views	Reconcile project developers’ worldviews with local people and other stakeholders during the design phase of the project. Reduce invisible powerlessness [individuals considering themselves less worthy of participatory opportunities] during project implementation	Wood et al. (2016, 2018)
Establishing independent grievance procedures	Independent grievance procedures can identify causes of procedural injustice not included in power analyses. Robust mechanisms need to be tailored to local specific conditions	Wood et al. (2018)
Challenging supra-local drivers of vulnerability	Projects to form part of wider social movements for change. Where resources are limited, umbrella organisations can draw on project experiences and co-ordinate appropriate responses	Hickey and Mohan (2005), Wood et al. (2018)

Procedural justice considerations may be especially relevant to BCEs. These environments often experience contested or absent tenure and overlapping jurisdictions between sectors such as forestry and fisheries. Around half of the small-scale fishers that rely on them are women, and fishing communities are often marginalised and poor, with seasonal flows of migrant workers and conflict between small and large scale sectors (FAO 2018). These factors will increase the chances of disadvantaging already marginalised groups during stakeholder conversations if procedural justice questions are not made explicit.

#### Theme 4: Land rights and tenure

##### What tenure and land rights do we need to ensure local communities have ownership of blue carbon ecosystems?

Inadequate or insecure tenure and property rights are recognised as a longstanding barrier to community-based natural resource management (USAID 2006; Lockie 2013), especially for blue carbon programmes (Hejnowicz et al. 2015; Beeston et al. 2020; Bryan et al. 2020). The allocation of tenure or property rights is often complicated on the coast, where ecosystems are typically common pool resources governed under different and overlapping sectors (Vanderklift et al. 2019). For example, responsibility for mangrove management is frequently shared across government ministries, often with conflicting mandates (e.g. Friess et al. 2016; Banjade et al. 2017). This complicated picture is likely to worsen as boundaries begin to shift landward through sea level rise (Sefrioui 2017). Furthermore, traditional customary (de facto*)* rights frequently coexist with formal *de jure* rights, particularly in Africa and the Pacific. Collectively, this results in a complex patchwork of property right regimes (e.g. public, private, common and open access), and institutional and legal mandates (USAID 2006; Olander and Ebeling 2011; Chimhowu 2019).

Some countries are now working towards including mangroves in forest definitions to be incorporated in NDC submissions. This may help resolve tenureship conflicts. An example comes from Tahiry Honko in Madagascar, in which conservation of 1200 ha of mangrove is supported by linking mangrove protection with the national REDD + strategy and selling carbon credits on the voluntary market (Rakotomahazo et al. 2019). In other cases, ownership rights to resources (such as the trees or the carbon they contain) rather than to land can be a route through tenureship barriers. A clear right to carbon is a requirement of accreditation in the voluntary carbon market (Bell-James 2016); many countries now have legislation that can, in principle, permit community tenureship of blue carbon (for example, the Tanzanian Forest Act (2002) and the Kenya Forest Act (2005)).

Formalising rights, however, does not guarantee fair outcomes for local communities. Experience with REDD + has shown that changes in carbon rights can lead to land grabbing and the exclusion of traditional landowners (Bryan et al. 2020). Hence, processes of formalisation should be founded on principles of deliberation, community partnership and co-production, and should avoid entrenching historical inequalities and setting-up new ones, whilst recognising customary (and historical) rights to resources (Rotich et al. 2016).

#### Theme 5: Communication and dissemination

##### How do we scale up local efforts and make it easier for communities to conserve and enhance blue carbon?

Local BCE projects may not make substantial contributions at the national scale. Scaling such projects and developing programmatic rather than project-based interventions will often be needed to make substantial contributions to mitigation and adaptation goals. Barriers to achieving this include policy, financial and technical challenges (Macreadie et al. 2019).

For instance, it has been recently highlighted that Small Island Developing States (SIDS) require greater multilateral collaboration to speed up national BCE assessments (Delgado-Gallego et al. 2020). Similarly, whilst Kenya’s NDC now has an explicit commitment to ‘conduct blue carbon readiness assessment for full integration of blue carbon/ocean carbon into NDCs’ (Tobiko et al. 2020), 70% of the estimated budget needed to achieve this must come from international sources. A tension exists between the importance, emphasised in themes 1–3, of careful, inclusive, and site-specific work with communities and the imperatives of enhancing impact at national and international levels. Resolving this requires proper investment in community engagement across thousands of sites, combined with more streamlined incorporation of these site-specific initiatives into national and international frameworks; traditional agricultural extension and outreach services, provided for decades in many countries, give multiple examples of how local changes can bring national results (FAO). Another example of direct relevance comes from the Forest Carbon Partnership Facility, which has operated since 2008 to help build national capacity for REDD + and has strong representation of indigenous people and civil society in its governance. Combining the science of NbS with collective action by bodies supporting initiatives such as the *Global Mangrove Alliance*, the *Blue Carbon Initiative*, the *Nature4Climate* and the *Bonn Challenge* can help to scale up local efforts for the inclusion of BCEs into upcoming NDCs, which increase in ambition with each submission (UNFCCC 2018).

### Policy and finance

#### Theme 6: Policy interactions

##### To what extent can blue carbon projects help meet NDCs for each nation?

If a country’s previous NDC has not referenced blue carbon, integration of blue carbon into the NDC should begin by checking any current relevant policies, followed by an assessment of the mitigation/adaptation potential of these nd how they can be promoted (Durham 2020). Box 1 provides an example of how Kenya has been supported in blue carbon inclusion for their NDCs.

Box 1 Case study: supporting Kenya in blue carbon inclusion in NDCs There was no reference to blue carbon, or indeed coastal or marine management for mitigation or adaptation, in Kenya’s first NDC submission in 2015, despite the country hosting two of the world’s few certified blue carbon projects (Mikoko Pamoja and Vanga Blue Forest), as well as having 455.9 km^2^ of mangroves (Kirui et al. 2013) and 317km^2^ of seagrass (Harcourt et al 2018), totalling carbon stocks of ~ 66 Mg (Cohen et al. 2013; Gress et al. 2017). The 2021 revision was developed by the Ministry of Environment and Forestry in consultation with a range of stakeholders, including the Kenya Marine and Fisheries Research Institute (KMFRI) and the Association for Coastal Ecosystem Services, organisations integral to the current Blue Carbon projects. The revised document now includes explicit commitments to BCEs and identifies options to meet these. For example, the Kenyan National Mangrove Ecosystem Management Plan was developed in 2017, but implementation has been slow; the explicit endorsement of this plan in the new NDC will encourage operationalisation. The opportunities for PES and other forms of new financing are noted, a full assessment of Blue Carbon to allow complete integration into the next NDC is anticipated and the document includes an explicit commitment to enhancing community governance in participatory resource management in coastal ecosystems

Having assessed current blue carbon opportunities and the scope for expansion, countries can access opportunities to participate in bilateral and multilateral agreements that support increasing the ambition of a country’s NDC targets. For example Internationally-Transferred Mitigation Outcomes (ITMOs) can help expand from the project level to the national level in order to over-achieve sector-specific targets. In some cases, Blue Carbon may make important (although never overwhelming) contributions to a nation’s NDCs. For example, Zeng et al. (2021) combine estimates of vulnerability to deforestation with assumptions about carbon prices to conclude that ~ 20% of the world’s mangroves could be conserved through carbon finance. In countries with large areas of mangrove and fast rates of loss, conserving these ‘financially viable’ sites could be important parts of NDC strategy; for example in Indonesia such sites could contribute ~ 1.8% of total NDCs. Of course, larger contributions could be made provided alternative resources, or policy changes, are made that conserve mangroves that are not ‘financially viable’ under these assumptions.

**Fig. 5:**
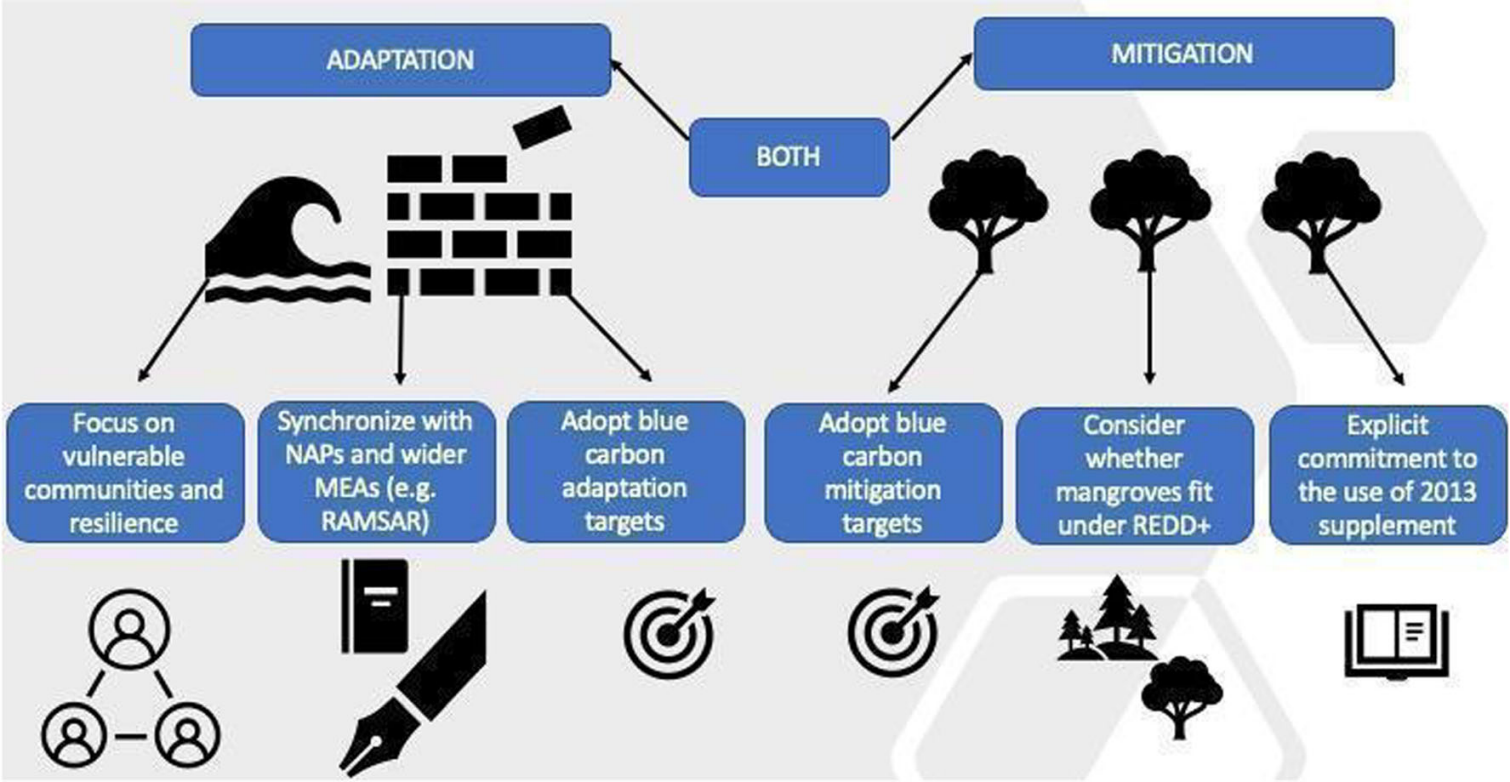
Decision tree for blue carbon in NDC with current adaptation and mitigation options for countries, adapted from Durham et al. (2020)

There should be clear alignment with existing and complementary national initiatives. For example, when developing strategies for inclusion of blue carbon in NDCs, the management and monitoring that accompanies those commitments should be aligned with any existing National Action Plans (NAPs) and Sustainable Development Goals (SDGs). This can reduce duplication of efforts and unnecessary complexity (Fig. 5).

**Theme 7: Markets and accounting**

**How will the Voluntary Carbon Market (VCM) tie into the NDC process and benefit local communities?**

The Voluntary Carbon Market (VCM) involves individuals, companies or organisations choosing to buy offsets as part of their carbon management strategies and has enabled several community-led blue carbon projects to access carbon financing. The role that the VCM will play (alongside the compliance carbon market, in which legally mandated offsetting takes place) is changing with the development of Article 6 of the Paris Agreement. Draft Article 6 texts, still under negotiation as of August 2021, do not explicitly mention the VCM, leading to uncertainty amongst VCM stakeholders on how the voluntary market will align with the compliance market and NDCs once Article 6 has been finalised. There are already hints that countries may want to prioritise NDC targets over independent carbon projects; Indonesia recently cancelled all ‘self-declared’ carbon projects in favour of directing carbon benefit towards national targets (Foresthints 2021).

A key uncertainty for community groups concerns the ownership of the carbon benefits generated by their activities. At COP26, the Article 6 ‘rulebook’ was finalised which includes mechanisms for credits to be ‘correspondingly adjusted’ if they are to be counted by a country other than the host country, in which case the VCM buyer can claim this offset and the host country adjusts their own accounting to reflect this transfer. Alternatively, credits that are not correspondingly adjusted can be sold on domestic markets or non-offsetting claims can be made by those who finance their production (i.e. a non-offsetting claim). This solution provides the transparency and credibility that is required as NDCs become more comprehensive and enhanced certainty for investors in carbon projects.

The draft Article 6 texts propose that a share of the revenue from trading on the new ‘sustainable development mechanism’, the successor to the Clean Development Mechanism, and potentially also from ITMOs, will contribute to a centrally administered ‘adaptation fund’ to assist developing nation Parties to meet the costs of adaptation. This may present an opportunity for the protection and restoration of BCEs not currently eligible for or included in carbon trading schemes.

Community-led BCE conservation and restoration projects contribute towards SDGs, biodiversity framework targets, climate change adaptation and disaster risk reduction efforts, in addition to carbon goals (UNEP 2020a). Recognising these broader contributions will help ensure community groups are supported under the implementation of NDCs. A range of alternative outcomes, in addition to carbon, are already recognised by the voluntary standards and indeed are implicit in their validation processes. Standards are now developing new forms of accreditation (for example ‘SDG credits’) to make these benefits explicit to buyers. If Paris Agreement goals are achieved, the relevance of current models of voluntary carbon offsetting will diminish as consumers change their lifestyles and threatened carbon-rich ecosystems are incorporated into and protected by strong national and international policy. The VCM can and should facilitate this transition whilst looking for new ways to achieve long-term sustainability. For example, IPCC models show that reducing emissions is not enough to achieve the 1.5 °C target; we must also address legacy carbon and continue to remove excess greenhouse gases from the atmosphere beyond 2050. The VCM can help develop the mechanisms to allow individuals and organisations to contribute to this legacy challenge.

**Theme 8: Funding**

**How do we reduce the cost of verifying blue carbon mitigation outcomes in small projects?**

Insufficient funds have limited the development of projects that conserve and restore BCEs under carbon standards (UNEP 2020b), with successful projects requiring considerable upfront funding which cannot always be recouped through carbon trading at current prices. Under current conditions only ~ 20% of mangrove forests present financially viable opportunities for blue carbon initiatives (and this figure is reduced to ~ 10% over a 30-year period; Zeng et al. (2021)) and so carbon trading is not a universally appropriate solution.

If blue carbon projects are to be developed at a scale to meet NDC targets, implementation costs must be reduced. National or international supported ‘hubs’ to guide communities and project developers through the accreditation and validation processes, and to share knowledge and resources to avoid doubling-up between projects, could help to achieve this. Involvement of government agencies in these national hubs will extend knowledge and resource sharing to encompass both NDC-level interventions and their associated policy implementation frameworks, and individual projects.

Many of the attractive features of Blue Carbon VCM projects arise from how carbon (and other ecosystem services) are ‘embedded’ in local institutions, livelihoods and ecosystems, with the acknowledgement of local context and agency leading to higher satisfaction amongst residents and buyers (Huff 2021). However, market and policy discourses that demand technical precision, certainty and fungibility only support ‘robust’ and repeatable carbon accounting, universal approaches and risk avoidance; effectively ways to ‘disembed’ the traded commodity of carbon or other services. This escalates costs (by for example demanding expensive techniques to measure carbon flows) and serves to exclude community involvement. The Blue Carbon community (and VCM project stakeholders more generally) needs to emphasise the already strong scientific and policy case for most examples of Blue Carbon conservation and restoration (particularly for mangroves), without exhaustive new justifications expected for every small, additional site. Cheap proxies for carbon (such as using aerial coverage to estimate below-ground storage), combined with appropriately conservative assumptions, can and should be acceptable in annual accounting.

### Science and technology

#### Theme 9: Ecosystem-based management

##### What are the knowledge gaps that hinder the inclusion of all blue carbon ecosystems in NDCs?

Data on the extent and dynamics of BCEs are often lacking, with large variations between nations in the quality of information. In recent years, significant progress has been made to increase knowledge on the carbon storage and sequestration potential of mangroves; seagrass and tidal marsh carbon storage and sequestration are less well understood, and this contributes in part to their current absence from carbon trading (Hejnowicz et al. 2015; Shilland et al. 2021).

There are nine BCE projects currently registered with the VCM (under Plan Vivo—two Kenyan and one Malagasy; under VCS—in China, Myanmar, Senegal, India and Indonesia; under Gold Standard—in India). All are based on mangrove conservation and restoration, with no examples of saltmarsh or seagrass meadow carbon trading projects. This limited uptake of carbon financing as a mechanism for blue carbon management is due, in part, to data gaps that hinder carbon calculations and present a barrier to certification (Wylie et al 2016; Shilland et al 2021). Similar problems apply to inclusion of BCEs within NDCs with many governments unaware or uncertain about the extent of their Blue Carbon resources and the options available to restore or protect them. This can be solved through strengthening of national blue carbon monitoring initiatives, without considerable increase in funding requirements, by linking them to national forest monitoring and REDD + MRV actions.

Of the three BCEs, seagrass meadows are both the most extensive and the most poorly understood. Scientific uncertainties limiting policy progress include i) the provenance of allochthonous carbon (produced outside of the seagrass ecosystem) and whether this can be claimed as carbon benefits by projects focussed on seagrass; ii) rates of carbon loss from seagrass sediment following damage or destruction, or rates of carbon accumulation following restoration or protection; and iii) the relevance of calcification in seagrass ecosystems, and of accumulation of inorganic carbon particularly carbonates, in calculating net carbon fluxes (see UNEP 2020a for an expansion of these scientific challenges). In addition, the total area and recent trends in coverage of both seagrass and salt marsh remain poorly known in many countries and regions. Whilst new developments in remote sensing are rapidly improving understanding of total coverage (see e.g. Mcowen et al. 2017), the other uncertainties will probably remain obstacles for policy at least in the near future. If salt marsh and seagrass are to be routinely incorporated into the next round of NDCs their roles in adaptation, as well as their contributions to other ecosystem services, need to be recognised; a narrow focus on carbon may continue to exclude them. Conservative assumptions on sequestration along with flexibility in crediting (for example by recognising additional outcomes such as contributions to SDGs) should allow inclusion of seagrass and saltmarsh into both VCM standards and NDC processes (Shilland et al. 2021).

#### Theme 10: Technology and methods

##### How do we simplify carbon accounting and validation methodologies so that they can be employed or contributed to by communities?

The IPCC Wetland Supplement (IPCC 2013) reports methodologies for mangrove, saltmarsh and seagrass management using three tiers. Tier 1 provides default emission factors for all relevant management activities, which can be used if nationally relevant emission factors are unavailable. Adopting Tier 1 emission factors provides the simplest route for community engagement with NDCs. For mangrove and saltmarsh management activities, including restoration, this requires monitoring changes in cover (area) and above-ground biomass and subsequently applying a number of default allometric equations. Determining large-scale changes in biomass of mangroves requires access to satellite or UAV (‘unmanned aerial vehicle’ or drone) imagery (e.g. Sanderman et al. 2018; Navarro et al. 2020), or can be done using field measurements on a smaller scale (Kauffman and Donato 2012). Provided training and support are available, such approaches are well suited to community engagement (Danielsen et al. 2011). Simplified methodologies not only streamlines monitoring, making it more coft-effective, but improves the accessibility of community engagement with the project, increasing equity and perceived legitimacy (Wells et al. 2017). Where there is the institutional support to identify relevant sites, provide training and ensure data analysis and reporting, activities for reversing mangrove forest removal and degradation or facilitating restoration provide a good opportunity for community engagement and one in which technical challenges are not the main barrier.

Only methodologies for the management activities of extraction and restoration are available for seagrass meadows (IPCC 2013). Seagrass restoration can be achieved by active replanting or reseeding, with the former practice being more expensive and requiring skilled direction. The major threat to seagrass meadows is from eutrophication and/or increased turbidity (Orth et al. 2006), often driven by changes in river catchments or coastal developments. As yet, not enough data are available to provide a methodology to account for the effect of these offsite activities. There has been an acceleration of research on BCE since the development of the 2013 IPCC Wetland Supplement and there is scope for new methodologies to be developed for other management activities that impact on seagrass meadows (Oreska et al. 2020).

Community engagement with Blue Carbon projects distinct from NDCs (such as validated VCM projects) present similar technical challenges, some of which are discussed in themes 8 and 9. As noted there, an explicit focus on benefits in addition to carbon may open up flexibility in the accreditation of projects under VCM standards. Standards that currently support BCE projects include the Verified Carbon Standard (VCS) and Plan Vivo. The former has approved a specified methodology, VM0033 (Verra 2019), which includes methods to assess abatement with restoration of mangroves, saltmarsh and seagrass meadows (Needleman et al. 2018). This methodology is generally at least as technically demanding—and in some cases much more demanding—than IPCC reporting requirements. Plan Vivo has incorporated mangrove projects using a case-by-case approach with Tier 1 assumptions permitted when deemed appropriate by their Technical Advisory Committee.

Whether the accounting of carbon benefits involves smaller local projects or is a combination of national and local scales, it is likely to remain a complex process involving multiple stakeholders and institutions. Some of this complexity may derive from the scientific processes needed for measuring carbon, but also from the needs to identify sites, demonstrate additionality and establish robust reporting. New risk measuring tools such as CORVI (Climate and Ocean Risk Vulnerability Index) and the IUCN Restoration Opportunities Assessment Methodology ROAM (IUCN 2021) may aid in the prioritisation and rapid assessment of coastal landscapes for blue carbon conservation. Whilst the development of new models, sensors, remote sensing techniques and computation tools to track blue carbon abatement may help streamline site identification and monitoring (Sani et al 2018; Navarro et al 2020), these approaches will require specialist skills and training.

In conclusion, barriers to community engagement arising from the technical and scientific challenges of carbon accounting may be lowered in some areas, through increased flexibility, robust models and consideration of broader outcomes in VCM routes to accreditation. However, the complexity inherent in the broader process remains a barrier for communities in many nations. Communities will continue to need supportive partnerships with government, NGOs and other sectors to be fully engaged.

## Synthesis and ways forward

Robust science shows that BCEs have exceptional value, not only for carbon but for a wide range of services. Inspirational projects demonstrate how coastal ecosystems can be conserved and restored for the benefits of nature and people. Growing international momentum promises ways of accelerating the conservation and restoration of BCEs, using climate change policy and other drivers. Realising this promise will require a collective effort to address the questions articulated here; we suggest three broad responses are needed, falling into our categories of people, science and technology and policy and funding. Paying proper attention to matters of inclusion and justice requires genuine commitment to understanding local contexts and to developing trust (Dencer-Brown et al. 2021). Project developers (and the standards and frameworks that support them) need to focus on this, even if it means sacrificing some scientific precision or accountability to markets. Hence, where the science is robust enough to justify Tier 1 approaches, combined with simple conservative assumptions (as is the case in many mangrove systems), a lack of precision should not be used to hinder accreditation of projects or rejection of BCEs from national policy. Research attention needs to be focussed on those areas—such as extent and trends of seagrass and saltmarsh and drivers of total carbon stocks in these habitats—which remain genuine barriers to conservative inclusion of some BCEs into local projects and national plans. On policy and funding, national approaches should invest in community support and retain the flexibility necessary to incorporate existing local projects with a range of funding from private, public and multilateral sources. BCEs are locally unique, resilient, adaptable and defy simple categorisations; our approaches to conserve them should be the same.

## Supplementary Information

Below is the link to the electronic supplementary material.

Supplementary file1 (PDF 870 kb)
