# Supplementary material for: Integrating blue: How do we make nationally determined contributions work for both blue carbon and local coastal communities?
Source: Ambio. 2022 May 3;51(9):1978–93. doi: 10.1007/s13280-022-01723-1 (PMC9063623; doi:10.1007/s13280-022-01723-1)
Supplement: Supplementary file 1 — Supplementary file1 (PDF 870 kb) [file 13280_2022_1723_MOESM1_ESM.pdf]

Ambio

Electronic Supplementary Material

This supplementary material has not been peer reviewed.

Title: Integrating Blue: How do we make Nationally Determined Contributions work for both blue carbon and local coastal communities?

Authors:

Amrit Melissa Dencer-Brown, Robyn Shilland, Daniel A. Friess, Dorothée Herr, Lisa Benson, Nicholas J Berry, Miguel Cifuentes-Jara, Patrick Colas, Ellyn K. Damayanti, Elisa López García, Marina Gavaldão, Gabriel Grimsditch, Adam P Hejnowicz, Jennifer Howard, Sheikh Tawhidul Islam, Hilary Kennedy, Rahma Rashid Kivugo, Joseph KS Lang'at, Catherine Lovelock, Ruth Malleson, Peter I. Macreadie, Rosalía Andrade-Medina, Ahmed Mohamed, Emily Pidgeon, Jorge Ramos, Minerva Rosette, Mwanarusi Mwafrica Salim, Eva Schoof, Byomkesh Talukder, Tamara Thomas, Mathew A Vanderklift, Mark Huxham,

Appendix S1

Theme 1: Environmental and Social Sustainability

How do we ensure that carbon projects support long-term changes to the use of land and natural resources that will be maintained after the period when carbon finance is available?

How will projects ensure regenerative ecology, risk management and resilience at the local level?

How do we communicate the need for systemic political change, promoting blue carbon along with other sensible options?

How can governments and communities optimise the social and environmental benefits of conserving blue carbon ecosystems?

What mechanisms support national policy and institutional programs, in order to ensure the permanence of blue carbon sinks at the community level?

Are there conditions that can be adhered to so that commitments made are achieved even when faced with political changes within local governments?

How do we address local drivers of deforestation and degradation without undermining the livelihoods of local communities?

How will community involvement be ensured and allow the sustainability of local livelihoods?

What happens for Nature-based Solutions, including blue carbon, if international agreements such as the Paris Agreement fall apart?

What possibilities exist for developing large-scale transboundary blue carbon initiatives, particularly where interconnected mangrove-seagrass ecosystems cross administrative and jurisdictional boundaries? And, in these instances, how would NDCs be equitably determined between countries?

For SIDS and vulnerable communities, how can we adapt and mitigate against climate change effects such as SLR and ensure the longevity of projects for this?

Theme 2. Participation and Collaboration

What is the level of participation (for example during developing policy instruments and in the management of natural resources) for local communities when it comes to NDC policy implementation and development?

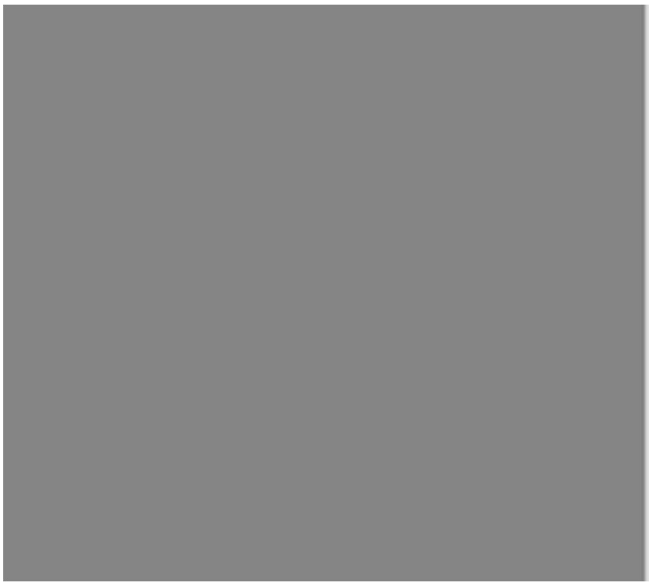

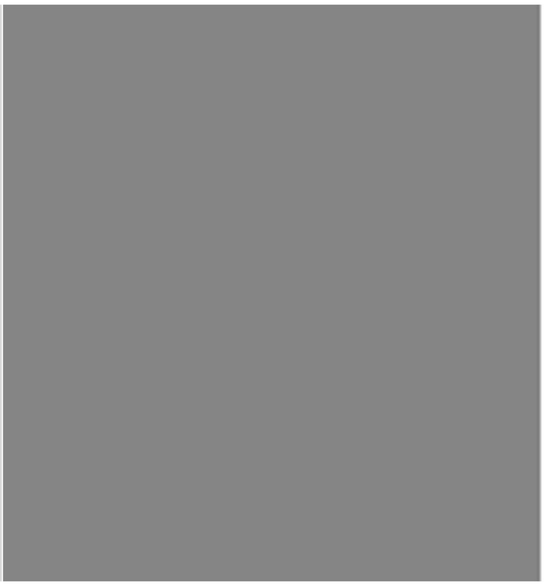

What are the most efficient and effective existing fora at local, district/county, national, regional level to discuss the role, representation, inclusion and contribution of community-based carbon projects in national and international NDC policies?

How will small and medium enterprises benefit in terms of human resource development when blue carbon is incorporated into NDCs?

What will be the participatory governance and institutionality required in our countries to ensure proper dialogues, co-planning and co-execution of community blue carbon projects?

How can governments, NGOs and community groups collaborate (nationally and internationally) to share resources, experience and skills required to implement blue carbon management at a local scale?

How do we promote greater participation of local communities in blue carbon projects (a mitigation issue) when their most pressing needs are related to adaptation, immediate livelihood needs, infrastructure, e.t.c?

How to make blue carbon conservation more financially and socially attractive than other alternative land uses and conservation approaches?

How do we ensure stakeholder mapping at the national level to create inclusive communication channels between authorities and the civil society, as well as encourage regional co-operation, for successful NDC implementation?

What would be the key enablers that could facilitate or encourage small landholders' participation in NDCs?
How can heterogeneity in communities within nations be incorporated into national strategies that meaningfully engage communities?

How can the buy-in at community level be obtained/assured and sustained?

Theme 3: Governance of local projects

How can credit and accountability for management successes and failures be distributed fairly in co-management between governments and community groups?

How can governments ensure that NDC targets are locally appropriate and acceptable among communities?

Can/how can capacity building among community groups/NGOs facilitate cost-effective implementation of NDCs?

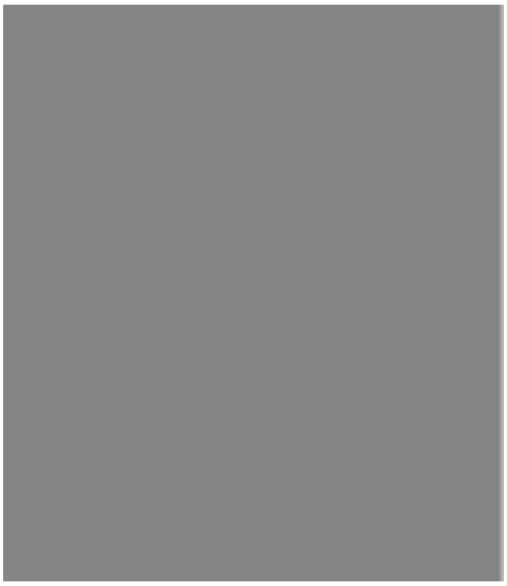

How can communities that have little access to information or know-how gain access to those organisations (i.e. GEF, World Bank, WRI, etc) that are funding activities which are directly or indirectly related to NDCs?

From a double-counting and marketing perspective are governments introducing safeguards to prevent impacting small landholders already engaged on emission reductions (i.e. Plan Vivo projects)?

To what extent are social justice concerns (e.g. gender and social equity) currently being built into the development of blue carbon initiatives, in relation to both the financial and ecological benefit flows received by communities, and how can these concerns be more central to the development of those initiatives?

Inclusion of Blue carbon into NDCs will limit extractive activities with an aim of increasing climate change mitigation efforts. How will the communities whose culture is tied to the blue carbon ecosystems, be supported so as to maintain their Business As Usual (BAU)?

What steps can be taken, drawing on past experiences, to ensure local, national and regional policy makers appreciate and value the role and contribution of communities and their indigenous knowledge in designing policy to encourage and enable (blue) carbon offsetting projects for the benefit of people and nature?

How can governments ensure that the vast majority of the profit/benefit from a BC project stays in-country and in the community?

Will the government provide support and funding to achieve the enabling conditions so the community will be in position to start implementing activities and deliver NDC targets?

How can we cost-effectively quantify the benefits of community-level carbon projects?

How can we distribute benefits from carbon projects within communities in a manner that benefits the most vulnerable and marginalised members of the community and avoids elite capture?

Where are the success stories that we can draw and disseminate lessons learned from and scale up/down from local to national initiatives?

How can we minimise unintended or perverse incentives associated with blue carbon? (e.g. blue carbon credits = more money in a community = investment in fishing gear = overfishing)

How can we make small projects count? (Remote sensing, inventories?) What mechanisms can be put in place to manage community expectations?

Theme 4: Land rights and tenure

How can land tenure rights be given to communities for sustainable management of resources but with contributions towards adaptation/mitigation going to the state?

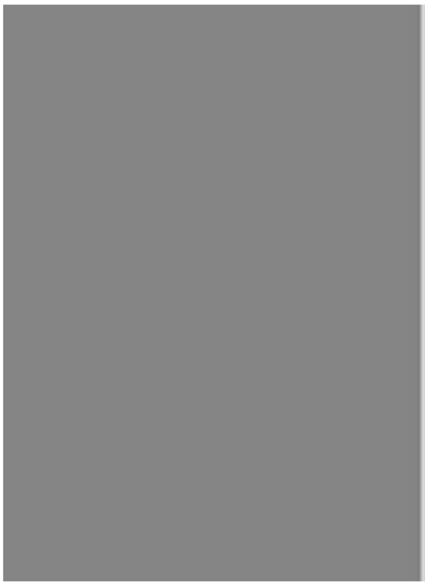

What tenure and land rights do we need to ensure local communities have ownership of blue carbon ecosystems?

Does the area and type of BCE have legal recognition by the national government as being community owned or managed?

Where are NDC targets from community-managed areas located in community/communally/privately-owned land or in state-owned land managed by community and who are the proponents of the targets?

Is there a risk that countries' NDC actions and commitments might disadvantage entire communities? For example, where reduction of deforestation and forest degradation is a national goal, there is a possibility that regulations might prevent community access and use of forest products.

How do we determine/clarify/claim land ownership so that carbon credits can be given directly to local coastal communities?

Theme 5: Communication and dissemination

How should we communicate the importance of blue carbon and Nature-based Solutions?

How do we scale up local efforts and make it easier for communities to conserve and enhance blue carbon?

Where can I learn about my country's NDC consultation process and can I directly provide input?

Where can I learn what sinks and sources my country includes in their national GHG inventory?

How do we ensure accurate awareness raising and education about these systems are available to communities to ensure conservation?

How can projects create awareness of the importance of carbon (in particular blue carbon)?

What are the governance processes for the project that involve multi stakeholders (community, business, NGOs, government ministries, etc.) and how will the project outcomes be captured and disseminated among stakeholders?

What lessons and good practice can be learnt and incorporated from previous relevant experiences (e.g. convention on biological diversity (CBD)) on access and benefit sharing ?

Is there political awareness of the adaptation and mitigation potential of the national Blue Carbon Ecosystems (BCE)?

Are there enough step-by-step and easy to follow community manuals and guidelines in establishing blue carbon projects? (including restoration manuals and other management

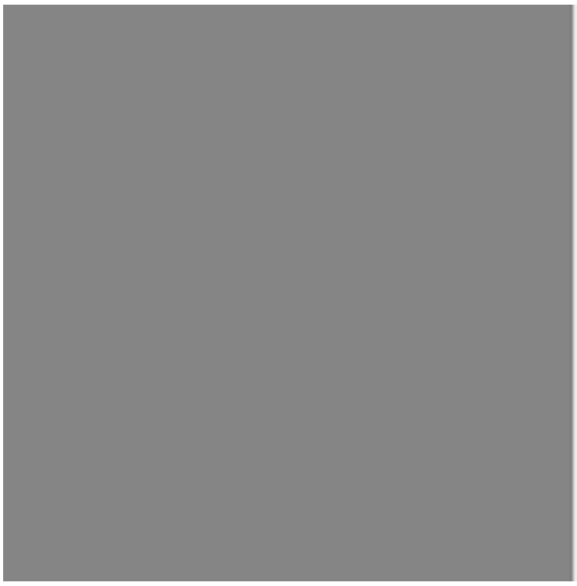

toolkits). If so, what hinders communities from adopting these manuals and establishing projects?

Are there systems in place to facilitate knowledge sharing, collaboration and cooperation to encourage broader transboundary projects that can be useful?

If blue carbon projects are transboundary, how do they contribute to each country's NDC? Are there community-driven initiatives that can be mainstreamed into NDC strategies?

Theme 6: Policy interactions

Can we establish RAMSAR-like protection systems internationally to protect important and vulnerable blue carbon assets?

To what extent can blue carbon / biosequestration projects help meet NDCs for each nation?

How can we integrate climate (UNFCCC) and biodiversity (CBD) goals through blue carbon project design and implementation?

How do we link the monitoring of blue carbon projects' mitigation targets transparently into larger forest cover monitoring, REDD+ and/or MRV systems while being consistent with national GHG reporting efforts?

How can BCE contribute to NDCs if relevant jurisdictions, governance, responsibilities and objectives overlap, conflict or are unclear?

How can we avoid moral hazard (i.e. using offsetting instead of reducing emissions) if blue carbon is part of international offsetting?

How are blue carbon sinks and capture going to be integrated into the National Emissions Inventory and how would emission factors be measured and standardised country-wide?

How is the blue carbon baseline measurement viewed from the federal and state government and from local capacities, how would their participation be encouraged?

Do NDCs consider just carbon sequestration/mitigation or is the need for adaptation and resilience for community-based projects considered at NDC level?

How do we ensure alignment with other international and national targets (Agenda 2030, National Adaptation Plan, National Mangrove Management Plan) and revise them to preserve community interests?

Theme 7: Markets and accounting

Is there a space for blue carbon outside of the voluntary market?

Can we incentivise carbon markets away from lowest-cost abatement, towards high-quality abatement?

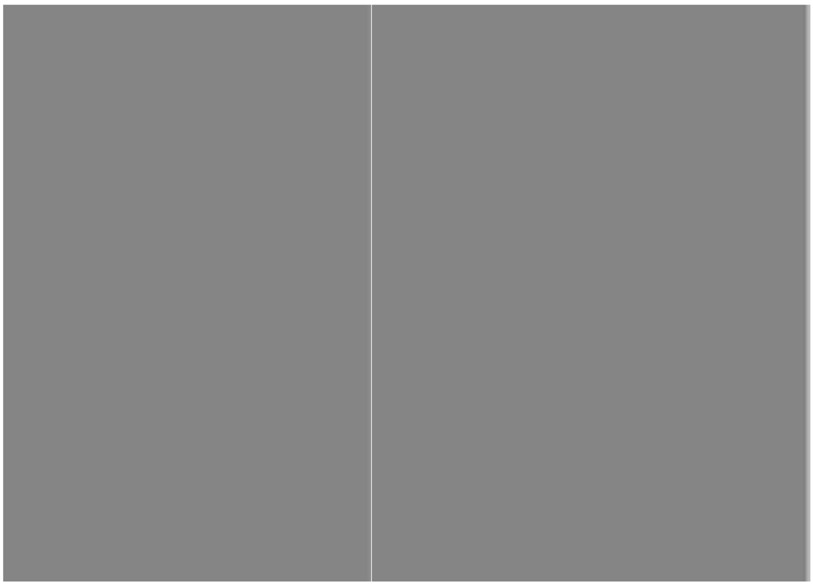

Will the carbon markets be enough to pay for the costs of blue carbon ecosystem restoration? Can we move carbon markets toward cryptocurrency (e.g. blockchain) platforms?

Can carbon markets be better geared to reward long-term carbon sequestration (e.g. blue carbon) over short-term sequestration (e.g. green carbon)?

How will the emissions avoided by voluntary markets be managed within national accounts?

What will be the mechanisms for the distribution of community benefits in the cases of PES or market schemes?

How do we secure income from the sale of carbon credits internationally without generating conflicts with the NDC through double claiming?

How will the Voluntary Carbon Market (VCM) tie into the NDC process and benefit local communities?

To what degree is it possible to buttress VCM trading credit value against market vagaries to guarantee a long-term stable dollar per volume amount for CO2 to ensure the longevity and sustainability of community-based blue carbon projects?

Will community-led projects be able to retain carbon rights for projects within the scope of NDCs?

Theme 8: Funding

How do we reduce the cost of verifying carbon mitigation outcomes in small projects?

Can we develop blended finance tools that can be used to underwrite the initial cost of developing small projects, and that are attractive to investors?

What policy and financing tools can be used to effectively implement NDCs at a community level?

If community-based/smallholder projects are incorporated into NDC accounting, will governments provide PES/results-based systems for communities and how will they be funded (e.g. could corporations provide funding and claim they supported the NDC of a country versus supporting individual projects)?

In the case of Kenya where blue carbon has not been incorporated in the NDCs, how do we set blue carbon as a priority and identify efficient financing means that will both directly and indirectly benefit the local communities?

If community-led initiatives are reframed under national contributions rather than voluntary markets where will the payment part of the PES come from?

How can we move away from carbon income as we go forward with the UNFCCC process? How can we find funding mechanisms to help local communities conserve blue carbon?

How can inclusion of blue carbon in the non-conditional NDC facilitate access to (voluntary) carbon markets and private investment?

Can blue carbon be used as a cost containment measure in a sectoral carbon pricing mechanism designed to meet the conditional NDC?

If voluntary community projects are not included in NDCs will the government allow communities to access non-government funds in order to get benefits from their activities, including from selling emission reduction certificates (enter into carbon offset agreement)?

Theme 9: Ecosystem-based management

How do we incorporate blue carbon ecosystems into broader governance and policy mechanisms for PES?

How can we definitely incorporate soil carbon and its spatial variability in carbon accounting schemes?

The focus on blue carbon projects is generally on mangroves, what constraints and opportunities are different for seagrasses?

What are the knowledge gaps in blue carbon ecosystems that hinder the inclusion of all blue carbon ecosystems in NDCs (methodologies in accounting for salt marshes, seagrasses and other potential ecosystems such as macroalgae/kelp)?

How do we move towards an ecosystem approach where we integrate different habitats? For example a combined mangrove – seagrass 'seascape' approach, including valuation of all ecosystem services (not just carbon).
How do we holistically incorporate different areas of ecosystem and projects into a country's NDC?

How can governments ensure that community-level drivers of degradation (e.g. need for firewood and timber) are addressed?

How do we best include relevant project-related activities in mangroves/seagrass conservation or restoration to raise national mitigation ambitions without ignoring adaptation measures?

What impact will the designation of protected areas and restricted zones for settlement have on the valuation of carbon, community livelihoods and the environment?

Are there existing clear aims and /or targets to strengthen or expand community BCE management in the country's NDC and if not is there the potential to include these?

Theme 10: Technology and methods

How do we simplify carbon accounting and verification methodologies so that they can be employed or contributed to my communities?

How do we prioritise and rapidly assess landscapes for blue carbon conservation?

How does science interface with the policy and legal frameworks to enhance incorporation of carbon into NDCs at a community level?

Is there a way of "pooling" credits from different projects and reducing certain transaction costs?

A lot of innovation around carbon methodologies and monitoring are coming out of community-based/smallholder projects - how can governments stimulate the same level of innovation and research?

Will a government accept pre-approved carbon methodologies at the project-level or would all projects have to use a nationally mandated methodology to feed into national-level accounting?

How will compliance with NDCs promote adaptation to climate change in communities and how could it be measured and reported (indicators)?

Are there technical as well as financial support systems in place to assist and encourage local communities in the development of blue carbon projects?

How can current gaps in monitoring and enforcement of environmental protection be filled in community-based management under NDCs?

What are the compliance mechanisms (monitoring obligations) for local communities (small landholders) to participate in the NDC process?

Where blue carbon systems are locally managed, what levels of capacity building, training and technology are required to enable local community actors to accurately determine and measure the carbon stock capacity of the interconnected mangrove-seagrass ecosystems under their management?

How can big data, machine learning and artificial intelligence be applied to more accurately model future carbon storage and sequestration potential of mangrove and seagrass ecosystems (taking into account the impacts of climate change, infrastructure developments as well as conservation initiatives) to determine the long-term influence on NDCs?

How can we support countries to adopt use of the IPCC wetlands supplement?
